# Adherence to a Mediterranean Diet and Impact on Mental Health Outcomes in Adolescents and Adults With Severe Mental Illness: A Systematic Review

**DOI:** 10.1192/bjo.2024.128

**Published:** 2024-08-01

**Authors:** Seetal Chavda, Amina Sarwar, Holly Batchelor-Parry, Kishan Pankhania, Rachel Upthegrove

**Affiliations:** 1 Coventry and Warwickshire Partnership NHS Trust, Coventry, United Kingdom; 2 Royal Free London NHS Foundation Trust, London, United Kingdom; 3 Birmingham Women's and Children's NHS Foundation Trust, Birmingham, United Kingdom; 4 Birmingham and Solihull Mental Health NHS Foundation Trust, Birmingham, United Kingdom; 5 Institute of Mental Health, School of Psychology, University of Birmingham, Birmingham, United Kingdom

## Abstract

**Aims:**

The Mediterranean diet has shown to improve mental health outcomes in people with depressive disorder. However, little is known of the impact of the Mediterranean diet on severe mental illness. The aim of this systematic review was to evaluate the impact of a Mediterranean diet on mental health and quality of life outcomes in adolescents and adults with severe mental illness (as defined by schizophrenia spectrum disorders, at risk mental states for psychosis, bipolar affective disorder and severe depression with psychosis).

**Methods:**

The following databases were systematically searched: MEDLINE and EMBASE via Ovid, CINAHL via EBSCO, PsychInfo via ProQuest, PubMed and Clinicaltrials.gov, using relevant subject headings and free text search terms to encompass severe mental illness and the Mediterranean diet. Screening, data extraction and quality assessment were completed by two independent reviewers. Eligible study designs included randomised controlled trials, other non-controlled or controlled interventional or experimental studies, cohort studies, case-control studies and cross-sectional studies that included adults and adolescents. The search was not limited to a specific time frame or language. The Mediterranean diet and mental health and quality of life outcomes were defined by primary paper author definitions.

**Results:**

Thirteen eligible studies were identified: 4 interventional, 2 cohort, 2 case-control and 4 cross-sectional studies and 1 mixed methods (interventional and observational) study. Diagnoses in most studies were psychotic illness, schizophrenia, schizoaffective disorder, bipolar affective disorder and depression with psychosis. There was a lack of studies found that included adolescents or participants with at-risk mental states for psychosis. A range of Mediterranean diet adherence scoring systems were used across studies, indicating a notable heterogeneity in the way adherence was evaluated. Most studies included other lifestyle exposures or interventions alongside the Mediterranean diet. There was a marked heterogeneity between studies in how mental health and quality of life outcomes were assessed. Although there was an overall trend towards improved mental health or quality of life outcomes in some studies, others reported no change or a negative association with the dietary/lifestyle exposure or intervention.

**Conclusion:**

The association between Mediterranean diet adherence and mental health outcomes and quality of life in adults and adolescents with severe mental illness remains inconsistent. Lifestyle-based interventions for the treatment of mental illness are cost-effective and relatively easy to implement with less concern about side effects. Therefore, this area requires further research.

